# Face Liveness Detection Using a Light Field Camera

**DOI:** 10.3390/s141222471

**Published:** 2014-11-27

**Authors:** Sooyeon Kim, Yuseok Ban, Sangyoun Lee

**Affiliations:** Department of Electrical and Electronic Engineering, Yonsei University, 134 Shinchon-Dong, Seodaemun-Gu, Seoul 120-749, Korea; E-Mails: sykim1221@yonsei.ac.kr (S.K.); van@yonsei.ac.kr (Y.B.)

**Keywords:** light field camera, face spoofing, face liveness, microlens image, sub-aperture image

## Abstract

A light field camera is a sensor that can record the directions as well as the colors of incident rays. This camera is widely utilized from 3D reconstruction to face and iris recognition. In this paper, we suggest a novel approach for defending spoofing face attacks, like printed 2D facial photos (hereinafter 2D photos) and HD tablet images, using the light field camera. By viewing the raw light field photograph from a different standpoint, we extract two special features which cannot be obtained from the conventional camera. To verify the performance, we compose light field photograph databases and conduct experiments. Our proposed method achieves at least 94.78% accuracy or up to 99.36% accuracy under different types of spoofing attacks.

## 1. Introduction

The face has been replacing personal identification number (PIN) codes and pattern locks due to its prominent features for human identification [1,2]. There is no risk of losing the face and there is no need to memorize passwords. For all those reasons, industries have applied face recognition technologies [3,4] to security systems in order to make those systems more convenient and stronger.

However, trials to invade security systems and duplicate personal information have occurred. Intruders abuse the weakness that machines cannot distinguish whether an input face is real or not. Current face lock technologies, indeed, cannot detect forged faces. This phenomenon shows the fatal error of the face recognition system. For this reason, technological defense against spoofing attacks is necessary to protect personal systems and users’ private data.

A lot of studies have been conducted to develop anti-spoofing technologies using visual cameras [5–32]. However, tools and skills for disguising identities also have been gradually evolved. To make defense systems stronger, researchers have considered using extra sensors as well as the visual camera. Thermal and near infrared sensors are some representative examples [14,33,34]. They can solve the vulnerability, but they do not have merits in terms of cost and commercialization. In order to satisfy these conditions, a novel method is proposed for detecting spoofing faces with a special sensor, light field camera.

The light field camera, developed by Lippmann in 1908 and evolved by Adelson and Wang [35], is a camera sensor that overcomes the limitations of the conventional camera. While the conventional camera outputs an image by summing all the lights falling into each pixel, the light field camera can record the direction as well as the color with the intensity of incident rays. In order that the light field camera saves the directions of the incident rays, a microlens array is embedded in front of the photosensor. Each microlens separates the light, and the direction and the intensity of the split light are recorded in pixels of the photosensor. Recently, commercial (or hand-held) light field cameras, such as Lytro [36] and Raytrix [37], are available in the market, and a camera array module that takes light field photographs in a mobile phone has been developed [38]. As a result, the light field camera is being applied in various research fields. Levoy [39] suggested a new method for generating 3D views from a light field. Kim [40] applied 3D light fields to reconstruct complex scenes in detail. With enough information of light fields, it is possible to reconstruct 3D scenes and produce super-resolution images [41,42]. Meanwhile, the light field camera is used for biometrics. Raghavendra *et al.* [43–46] showed the strength of the light field camera for face and iris recogntion. For multiple face recognition [44], they used depth images extracted from the light field. Also, in [46], they employed multiple focus images for iris recognition. Likewise, refocusing technology, the main characteristic of the light field photograph, is fully utilized to recognize biometric traits. However, in this paper, we will analyze the raw light field photograph microscopically and apply it to detect spoofing faces.

Raw light field photograph is composed of a lot of small images called as microlens images. Depending on the location of the focal plane, microlens images represent different light distributions. This helps to estimate the existence of depth in the input image. In this paper, by analyzing the properties of the raw light field photograph, we introduce new feature descriptors extracted from the raw light field photograph. In addition, an anti-spoofing face method is proposed applying new feature descriptors. To evaluate our method, we create databases using the light field camera and measure error rates in experimentation section.

The remainder of this paper is organized as follows. In Section 2, we introduce research about face liveness detection. Moreover, the light field photograph and its characteristics are mentioned. Our proposed method and the new features are stated in Section 3. In Section 4, the composition of databases and measures for evaluation are explained, and experimental results are shown. Finally, concluding remarks are provided in Section 5.

## 2. Backgrounds

### 2.1. Face Liveness Detection

**Figure 1.**
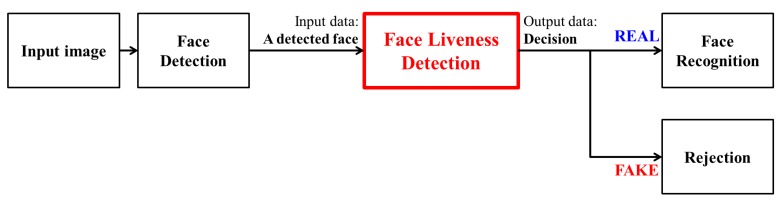
Face recognition system including face liveness detection.

Face liveness detection (or face spoofing detection) is a process to determine whether a detected face is real or not before a face recognition system identifies the face, as illustrated in Figure 1. It prevents the face recognition system from making a wrong decision. There are several types of spoofing faces, such as 2D printed photos, videos, high-definition (HD) tablets, 3D masks, and so on. Among them, 2D photos are used widely because they are easy and cheap to obtain. To minimize the vulnerability against 2D attacks, researchers have shown steady progress in developing anti-spoofing technologies based on features of 2D photos [5]. There are some characteristics in recaptured 2D photos. First, detailed components and sharpness are lost. In this case, researchers analyze texture and frequency components in the input data. In order to represent the textural feature, local binary patterns (LBP) are often used [6,7]. On the other hand, some methods detect high frequency components and look into power spectrum [6,8–12]. Although feature domains are different, those studies approach to the solution in terms of texture. Second, there is a difference in light distributions on a face. This approach focuses on skin reflectance of real and fake faces. Tan *et al.* [13] and Zhang *et al.* [14] utilize a Lambertian model to pull out the information for finding fake faces. Bai *et al.* [15] calculate specularity components from the input data. Huang *et al.* [16] perceive the change of artificial shadows on a face. Third, 2D spoofing faces have little motion, compared to real faces. Signs of liveness are perceived in image sequences of real faces, but not in those of fake faces. This can be a clue to distinguish motionless fake faces. Several research papers [17–19] concentrate on eye blink and movement. In addition, facial movements are helpful to analyze the differences between real and fake faces [20–24]. Komulainen *et al.* [29] suggest a countermeasure with the fusion of motion and micro-texture analysis methods. The last approach is based on 3D facial information. The obvious difference between a real face and a 2D photo is the presence or absence of depth information. Researchers have classified spoofing attacks by considering the depth feature. Lagorio *et al.* [25] and Wang *et al.* [26] present a solution based on 3D facial shape analysis. Kim *et al.* [27] propose a method using a difference between degrees of variable focusing.

Even though a lot of studies have been increasingly developed to protect facial security systems against spoofing attacks, tools and skills for disguising identities have been gradually evolved. In particular, masks and camouflages make it difficult to classify faces using above approaches. To tackle these problems, researchers have considered using extra sensors as well as a visual camera. There are typical studies using thermal and near infrared sensors. Zhang *et al.* [14] propose a method that measures the reflectance of skin using near infrared sensors. Sun *et al.* [33] show a thermal IR and visible light correlation system with a thermal infrared sensor. Kant *et al.* [34] present a real-time solution using a thermal image and skin elasticity of a human face. These suggestions can resolve the vulnerability, but they do not have merits in terms of cost and commercialization because of the usage of extra sensors. In order to exceed the limit, in this paper, we propose a novel method using a specific sensor, light field camera.

### 2.2. Light Field Photograph

**Figure 2.**
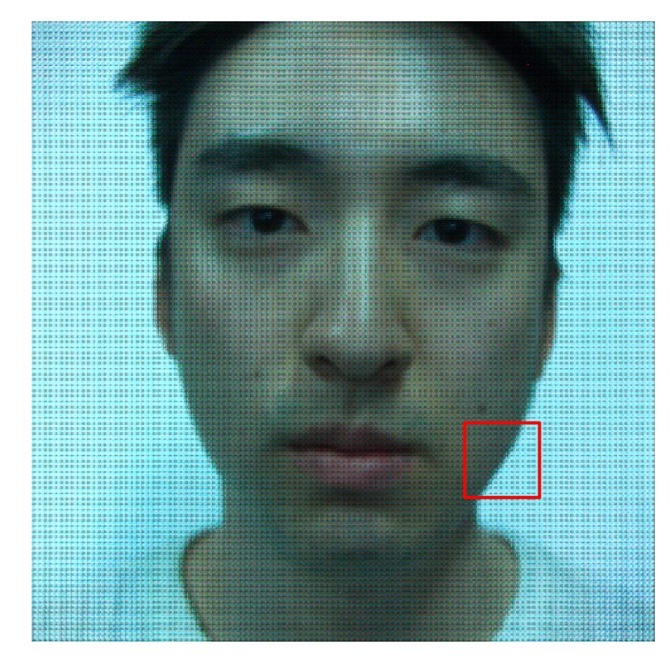
Raw light field photograph.

**Figure 3.**
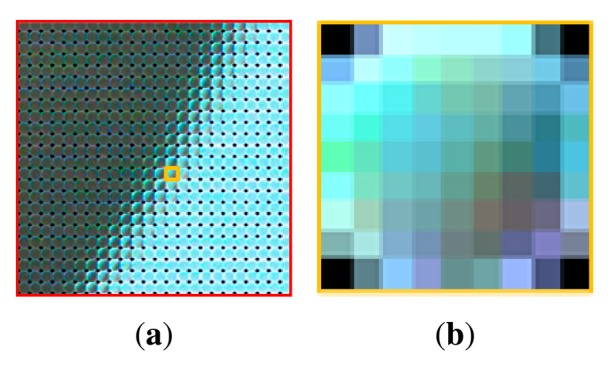
Analysis of raw light field photograph: (**a**) parts (red box) of Figure 2; and (**b**) one (yellow box) of microlens images.

As mentioned in Section 1, the light field camera is a sensor that can record information from incident light rays. The information of the light contains not only the intensity and color, but also the directions of the light rays. In this paper, we use a Lytro camera to capture the light field. A Lytro camera is the first consumer light field camera developed by R.Ng [36]. This camera makes it possible that users capture the light field at anytime and anywhere. In addition, users can import the light field from the camera into a computer and export light field photographs from the imported light field, using a software provided by Lytro Inc. [36]. The imported data is a LFP (Light Field Picture) file. LFP is a picture format that contains the captured light field. Each LFP file records 11 Megarays, but it is impossible to view and access to the file directly because it is 4D data. In order to visualize the 4D light field, we project the light field onto a 2D image. Lytro software makes it possible to view the light field image, but it has a limitation to access into the internal information of the light field data. Therefore, we use the open source (LFP reader program [47]) and the tool box (Light Field Toolbox for MATLAB [48]). By running the LFP reader program, we can obtain raw images (.RAW) and their associated metadata (.JSON). In order to analyze those data in MATLAB, we decode them using Light Field Toolbox. This tool box is introduced for the Lytro camera. With the Light Field Toolbox, the light field (*LF*) can be extracted. *LF* is a 5D array and its size is 9 × 9 × 380 × 380 × 4. The angular resolution of the Lytro camera is 9, and the spatial resolution is 380. Four is both the size of values of R, G, B color channels and the weight which represents the confidence associated with each pixel. Figure 2 is the result of decoding the original light field data. This image is called as raw light field photograph. Figure 3a shows the expansion of the raw light field photograph. As described in Figure 3, the raw light field photograph is composed of a lot of small circle images. These small images are called microlens images [49]. Figure 3b is one of the microlens images. Each microlens image shows the incident light ray that leaves from different positions and arrives at the photosensor through the microlens array. We will mention this image in detail in the following subsection.

#### 2.2.1. Two Views of Raw Light Field Photograph

Raw light field photographs have enough information about incident rays. In the following, we analyze two visualized images and their characteristics.

##### Microlens Image

Photosensor pixels are assigned to each microlens and form a small image. This image is referred to as the microlens image [49]. In the raw light field photograph, there are as many microlens images as the number of microlenses. For example, if the microlens array consists of 100 microlenses, there are 100 microlens images in the light field photograph. Each microlens image shows the incident light ray that leaves from different positions and arrives at the photosensor through the microlens array. There is a notable property in the microlens image. According to [50], microlenses at the focal plane have constant values in color because every incident ray originates from the same point on the focused subject. However, if the subject is not in focus, microlens images do not have constant values. When the microlenses are further than the focal plane, the light distributions inside the microlens images are inverted. The reason why this phenomenon happens is that the incident rays are inverted as they pass through the focal plane. More details are explained in [50].

**Figure 4.**
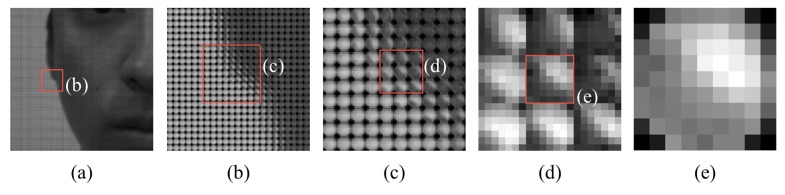
Raw light field photograph.

Figure 4 is our own sample of magnified views of Figure 2. The focal plane of the original light field photograph lies on the nose tip. Figure 4a is a region of the left ear and chin, and Figure 4b is the macroscopic image of Figure 4a. Figure 4e is the microlens image that lies on the edge of the ear, and Figure 4d illustrates adjacent microlenses of the microlens Figure 4e. Microlenses on the edge of the ear are farther than the focal plane. Therefore, the inversion of the incident rays occurs. The light distribution inside Figure 4e is opposite to the light distribution of the macroscopic image in Figure 4b. Depending on the location of the focal plane, microlenses can have different features. In this paper, we extract a feature descriptor based on this characteristic of the microlens image. We will mention it minutely in Section 3.1.1.

##### Sub-Aperture Image

**Figure 5.**
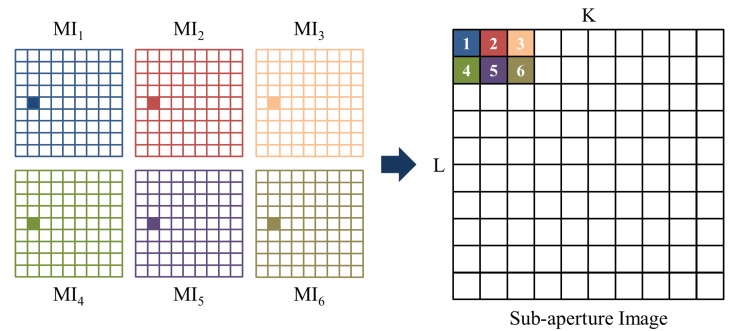
Process of making a sub-aperture image.

Sub-aperture images [49] are made by reordering incident rays in the raw light field photograph. Figure 5 illustrates the process of making a sub-aperture image. Each sub-aperture image is composed of the pixels of same position selected from each microlens image. According to the location of the pixel, multiview sub-aperture images can be obtained and have different information of incident rays respectively. The conventional photograph is equal to the integration of all sub-aperture images, summing all the incident light.

## 3. Proposed Methodology

In this section, we propose a method for face liveness detection by analyzing characteristics of the raw light field photograph. We suggest two feature descriptors extracted only from the light field photograph. In the following subsections, we will explain the processes of extracting features and classifying spoofing faces.

### 3.1. Feature Extraction

To detect fake faces, we propose two types of feature descriptors, edge and ray difference features. Edge feature is extracted from microlens images located on the edge of the lower jaw, and ray difference feature is from the difference between sub-aperture images. Details are explained in the following subsections.

#### 3.1.1. Edge Feature

**Figure 6.**
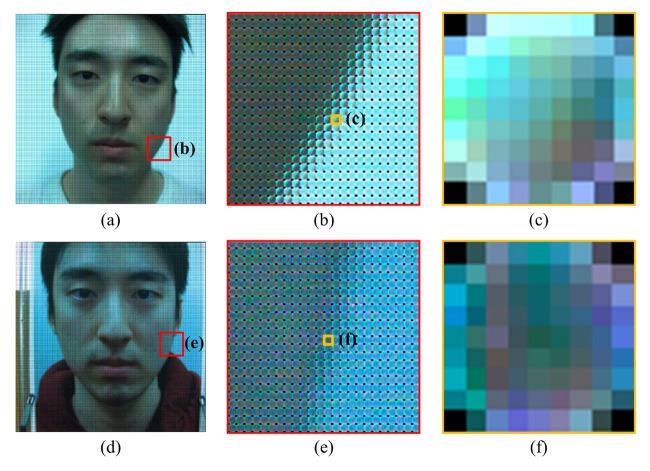
Light field photographs: (**a**) raw data of a real face; (**b**) magnification of real LF photo; (**c**) microlens image which lies on the edge in real LF photo; (**d**) raw data of a fake face; (**e**) magnification of fake LF photo; and (**f**) microlens image which lies on the edge in fake LF photo.

Edge feature is based on the characteristic of the microlens image. As mentioned in the previous section, microlens images have different distributions of lights, according to whether the corresponding area is on the focal plane or not. We focus on this property to classify real and fake faces. Real faces have a depth gap between the nose and ear areas. However, fake faces, such as 2D flat photos and warped photos, have relatively little difference between the depths of the nose and the ear. This feature is checked in the microlens images of the raw light field photographs. Figure 6a,d are raw light field photographs of real and fake faces. The focal planes of those pictures are on the nose. Figure 6b,e are microlens images near the chin, and Figure 6c,f are the examples of microlens images corresponding to the chin of the real and fake faces respectively. The microlens image of the real face (Figure 6c) is not full of lighting rays and has a gradational variation of pixel values as if there is a short edge. However, the microlens image of the fake face (Figure 6f) has randomly uniform distribution. Likewise, we can make it clear to distinguish real and fake faces by inspecting microlens images.

The light field photograph is composed of many microlens images. Through the procedure of decoding the light field [36,47,48], 144,400 (= 380 × 380) microlens images are obtained. The quantities of microlens images are so large that we cannot analyze all of them. Therefore, we pick out microlens images on the edge of the lower jaw and extract an edge feature from the selected microlens images.

**Figure 7.**
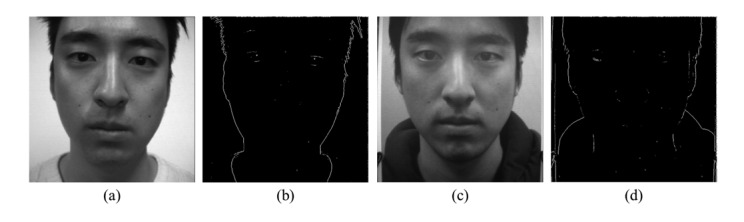
Edge detection from a sub-aperture image: (**a**) a sub-aperture image of the real face; (**b**) vertical edges of the real face; (**c**) a sub-aperture image of the fake face; and (**d**) vertical edges of the fake face.

**Figure 8.**
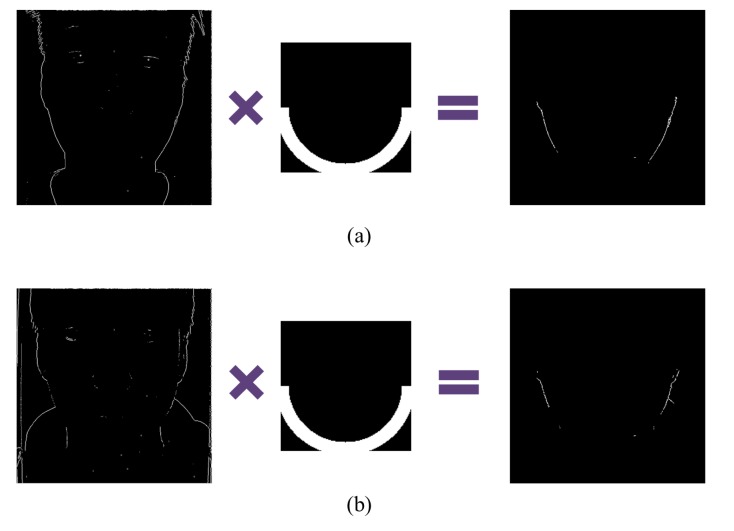
Masked edges of (**a**) the real face; and (**b**) the fake face.

In order to detect edges, we make a sub-aperture image using a center pixel in microlens images. Figure 7a,c are sub-aperture images of real and fake faces respectively. For vertical edge detection, a sobel filter is applied to the green and blue channel images whose noises are smaller than the noises in the red channel image. In Figure 7b,d are vertical edges of real and fake faces. Among extracted edges, there are irrelevant edges which may cause confusion in distinguishing fake faces. In order to filter unnecessary edges out, a semicircular mask is adopted to detected edges. Figure 8 shows results of masked edges of real and fake faces. Afterwards, we arrange microlens images corresponding to the selected edges. Through this process, we can choose microlens images which must be analyzed.

**Figure 9.**
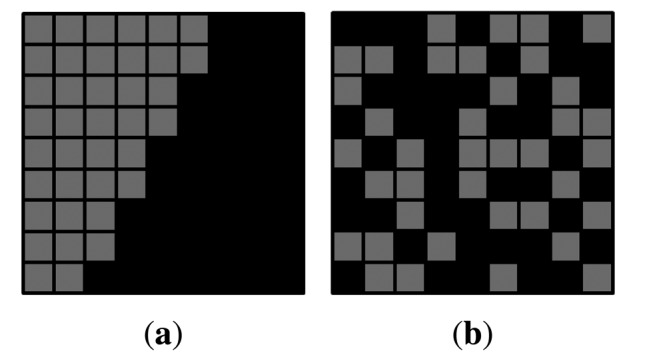
Examples of microlens images: (**a**) “0” and “128” clusters; and (**b**)“0” and “128” (randomly distributed).

As mentioned in the previous section, there is a change in light distributions of the microlens image located at the defocused edge. In order to inspect the lighting variation, we may calculate variances of microlens images. However, the variance of the entire microlens image is not a distinguishable feature. Figure 9 shows an example. There are two microlens images. Both Figure 9a,b have the same pixel values. However, Figure 9a is clearly divided into two regions, while Figure 9b has a random distribution of pixel values. Both variances of those images are same, but the appearances of them are different. Therefore, the variance of the entire area cannot become a criterion to discriminate various light distributions in microlens images. To solve this problem, we analyze the microlens image locally adopting a local binary pattern (LBP) [51,52].

We compute two types of binary patterns, inner binary pattern (*BP^in^*) and outer binary pattern (*BP^out^*). Inner binary pattern is extracted from the inside of the microlens image, and outer binary pattern is from the surrounding microlens images.

##### Inner binary pattern

**Figure 10.**
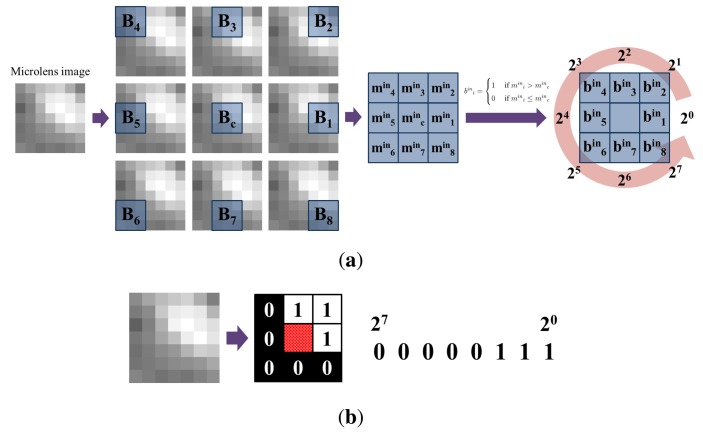
Inner binary pattern of a microlens image: (**a**) process; and (**b**) example.

Figure 10a describes the process of computing the inner binary pattern (*BP^in^*). The microlens image is split into 9 subregions, allowing the overlap between subregions. Although the size of the microlens image is 9 × 9, we use 7 × 7 region which is less sensitive to the lighting variation. *B_c_* is a center subregion and *B_i_*(*i* = 1, …, 8) are adjacent subregions. Each subregion is a 3 × 3 block, and we compute the average value of the subregion. *m^in^_c_* is the average value of the center subregion, and *m^in^_i_*(*i* = 1, …, 8) are those of the adjacent subregions. By the comparison of averages, a bit stream is yielded. If *m^in^_i_* is larger than *m^in^_c_*, the bit of the corresponding subregion, *b^in^_i_*, is 1. Otherwise, the bit is 0. Each microlens image has one bit stream, and each bit stream is composed of 8 bits. Equation (1) explains how to make the inner binary pattern. *px* means an intensity of each pixel. Figure 10b is an example of the inner binary pattern of the microlens image.

(1) $$\begin{array}{c} {b^{in}}_{i}=\{\begin{array}{cc} 1 & \text{if}\;{m^{in}}_{i}>{m^{in}}_{c}\\ 0 & \text{if}\;{m^{in}}_{i}\leq {m^{in}}_{c} \end{array}\\ {m^{in}}_{i}=\frac{1}{3\times 3}{\text{∑}}_{px\in B_{i}}px\\ BP^{in}=\left[\begin{array}{cccc} {b^{in}}_{8} & {b^{in}}_{7} & \ldots & {b^{in}}_{1} \end{array}\right] \end{array}$$

**Figure 11.**
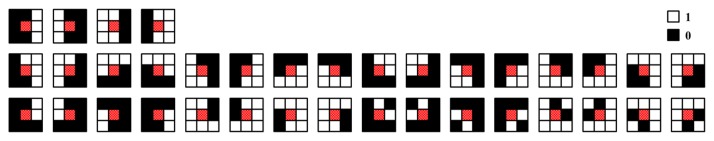
Edge patterns

According to light distributions, the microlens image has one of the 256 inner binary patterns. We categorize those patterns as either edge or non-edge based on the appearance of the pattern. Microlens images of concern are extracted from the edge of the chin. Therefore, we have an interest in the microlens images that have edge patterns. As described in Figure 11, 36 edge patterns are considered. Horizontal edge patterns are not applicable because we detect vertical edges in the previous step.

**Figure 12.**
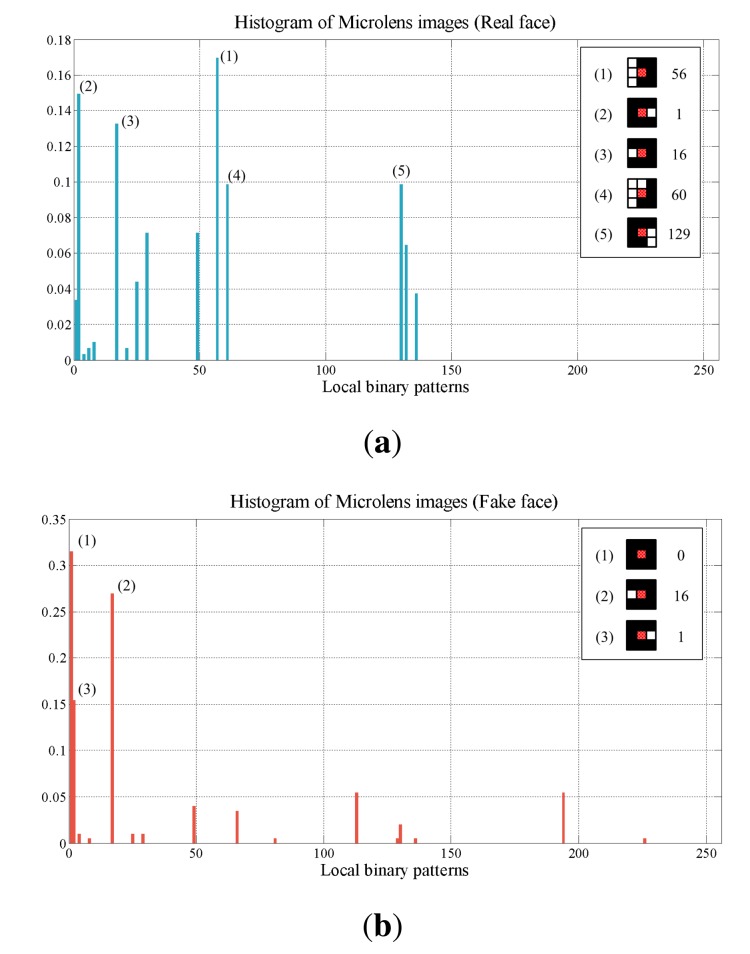
Histograms of inner binary patterns of (**a**) the real face; and (**b**) the fake face.

Figure 12 shows the histograms of inner binary patterns of the real and fake faces. Inner binary patterns of high frequency and these decimal numbers are also shown at the corner of those plots respectively. The real face has edge-shaped inner binary patterns, such as 56, 60, 129, much more than the fake face has. On the other hand, the fake face has more non-edge patterns than edge patterns. Based on this property, we focus on inner binary patterns corresponding to edge patterns.

##### Outer binary pattern

**Figure 13.**
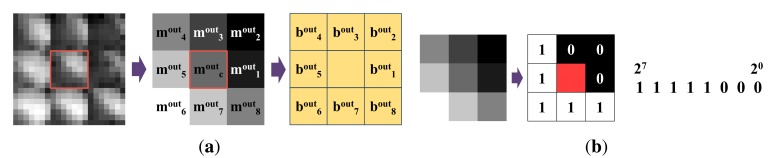
Outer binary pattern of a microlens image: (**a**) process; and (**b**) example.

Outer binary pattern (*BP^out^*) is made by comparing the microlens image with surrounding microlens images. The left image in Figure 13a represents microlens images. 8-neighbor microlens images are used. The middle image in Figure 13a is a set of averages of microlens images. *m^out^_c_* is the average of the center microlens image, and *m^out^_i_*(*i* = 1, …, 8) are averages of surrounding microlens images. When the average is computed, the 7 × 7 region in the microlens image is used. Like the inner binary pattern, the outer binary pattern is obtained through the comparison with averages of adjacent microlens images. Equation (2) represents how to make the outer binary pattern. *px* is an intensity of each pixel, and *MI_i_* is the *i_th_* microlens image. Figure 13b shows the outer binary pattern.

(2) $$\begin{array}{c} {b^{\text{out}}}_{i}=\{\begin{array}{cc} 1 & \text{if}\;{m^{\text{out}}}_{i}>{m^{\text{out}}}_{c}\\ 0 & \text{if}\;{m^{\text{out}}}_{i}\leq {m^{\text{out}}}_{c} \end{array}\\ {m^{\text{out}}}_{i}=\frac{1}{7\times 7}{\text{∑}}_{px\in MI_{i}}px\\ BP^{\text{out}}=\left[\begin{array}{cccc} {b^{\text{out}}}_{8} & {b^{\text{out}}}_{7} & \ldots & {b^{\text{out}}}_{1} \end{array}\right] \end{array}$$

##### Variance of the averages of subregions in the microlens image

Depending on the light distribution, binary patterns have different aspects. However, binary patterns are insufficient to conduct quantitative analysis of the lighting variation. Therefore, we calculate the variance of the averages of subregions (*m^in^_i_*) using inner and outer binary patterns. In Equation (3), *v_j_* is the variance of *m^in^_i_* in the *jth* microlens image, and *μ_j_* is the mean of *m^in^_i_* in the *j_th_* microlens image. *c_i_* is 1 or 0, and this is determined by inner and outer binary patterns. As mentioned in Section 2, due to the depth gap between the focal plane and another position further from the focal plane, the microlens image (Figure 4e) has the inverted light distribution, compared with the macroscopic image (Figure 4b). That is, the inner binary pattern is the reversal of the corresponding outer binary pattern. However, if there is little depth gap or the region of interest is closer than the focal plane, this feature is not valid. Therefore, in order to highlight the characteristic of the microlens image, *c_i_* is 1 as the bit of the inner binary pattern is the opposite of that of the outer binary pattern. Otherwise, *c_i_* is 0.

(3) $$\begin{array}{c} v_{j}=\frac{1}{7}{\text{∑}}_{i=1}^{8}{\left({m^{in}}_{i}-\mu_{j}\right)}^{2}c_{i}\\ \mu_{j}=\text{mean}\left(c_{i}{m^{in}}_{i},i=1,\ldots 8\right)\\ c_{i}=\{\begin{array}{cc} 1 & {b^{in}}_{i}\neq {b^{\text{out}}}_{i}\\ 0 & {b^{in}}_{i}={b^{\text{out}}}_{i} \end{array} \end{array}$$

We assign weight to variances in accordance with how many microlens images with edge patterns exist. The weight *w* is the ratio between the number of microlens images with edge patterns (*N_E_*) and the number of total microlens images (*N_M_*). If microlens images of edge patterns comprise a large proportion of the total microlens images, extracted features can be regarded as reliable data to determine the input face.

(4) $$\begin{array}{c} V=w\times \left[\begin{array}{ccccc} v_{1} & v_{2} & v_{3} & \ldots & v_{N_{M}} \end{array}\right]\\ w=\frac{N_{E}}{N_{M}} \end{array}$$

**Figure 14.**
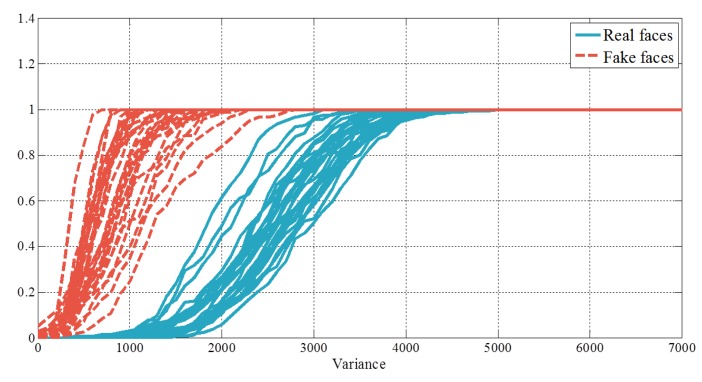
Cumulative distributions of variances.

**Figure 15.**
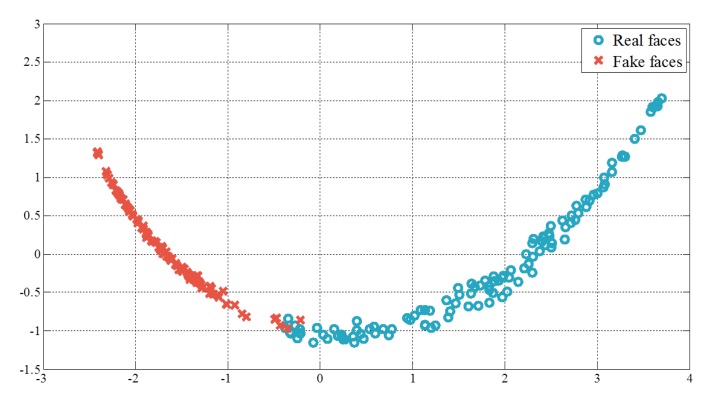
Principal component analysis (PCA)—Transformed features.

The number of the microlens images, chosen in the edge detection step, is different in every input data. Therefore, it is difficult to compare arrays of variances directly in case the lengths of arrays are not the same. To make a comparison among arrays of variances, we build histograms of variances and calculate cumulative distributions of those histograms. The histograms are normalized to 1. Figure 14 shows cumulative distributions of histograms. Blue solid lines present the results of real faces, and red dotted lines show those of fake faces. Variances of fake faces are massed in the lower region, but those of real faces are not. In the final step, principal component analysis (PCA) is applied to the cumulative distributions and their representative eigenvectors are found. By projecting the cumulative distributions onto those eigenvectors, we can obtain new features and utilize them as edge features. Distributions of the edge features are presented in the Figure 15.

Algorithm 1 shows the whole process of extracting the edge feature.



**Algorithm 1** Feature Extraction: Edge Feature
1:Make a sub-aperture image (Figure 7)2:Find vertical edges of faces in the sub-aperture images (Figure 7)3:Select corresponding microlens images using a semicircular mask (Figure 8)4:**for** Each microlens image *MI_n_*
**do**5: Compute an inner binary pattern (*BP^in^*) and an outer binary pattern (*BP^out^*) (Figures 10 and 13, Equations (1) and (2))6: Determine whether *BP^in^* of *MI_n_* belongs to the set of edge patterns (Figure 11)7: **if**
*BP^in^* belongs to the set of edge patterns **then**8:  With *BP^in^* and *BP^out^*, calculate a variance (*v_n_*) of the averages of subregions in *MI_n_* (Equation (3))9: **else**10:  Move to the next microlens image *MI_n_*_+1_11:Multiply a variance set *V* and a weight *w* (Equation (4))12:Make a histogram of *V*13:Make a cumulative distribution of the histogram (Figure 14)14:Apply PCA to the cumulative distribution (Figure 15)


#### 3.1.2. Ray Difference Feature

If there are few edges in the sub-aperture image, it is difficult to extract the edge feature for spoofing detection. In order to distinguish fake images without edge features, we propose the other feature called as ray difference feature. This feature is extracted from sub-aperture images. As mentioned in Section 2.2.1, the sub-aperture image is made by reordering pixels from microlens images. In the process of extracting the ray difference feature, we analyze sub-aperture images that have different information of incident rays and the difference between sub-aperture images of real and fake faces. Algorithm 2 shows the process of extracting the ray difference feature.



**Figure 16.**
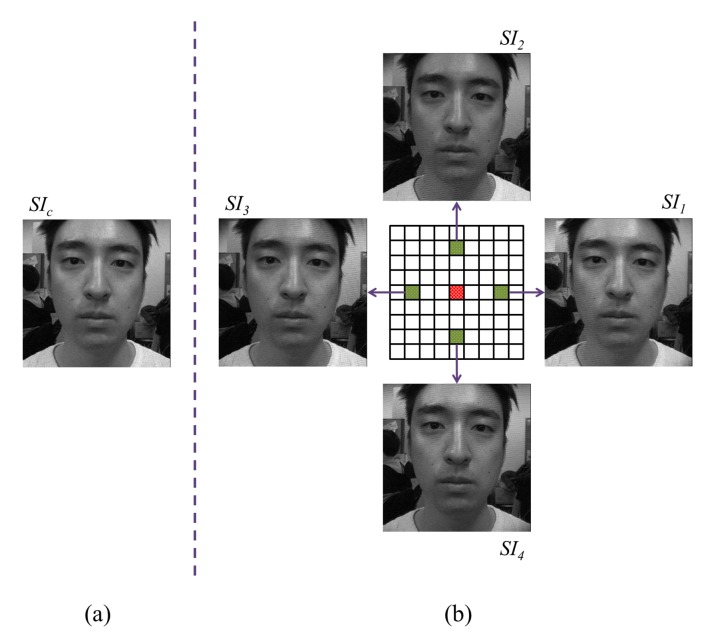
Sub-aperture images (**a**) from center pixel (5, 5); and (**b**) from neighbor pixels.

**Figure 17.**
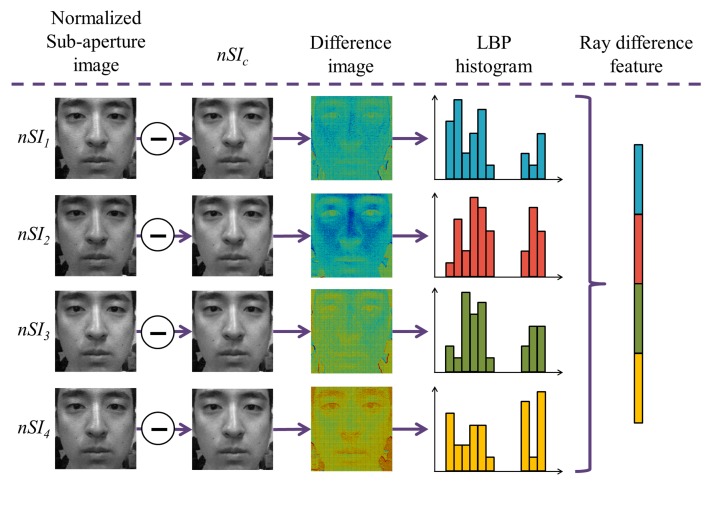
Process of extracting ray difference feature descriptor.

**Figure 18.**
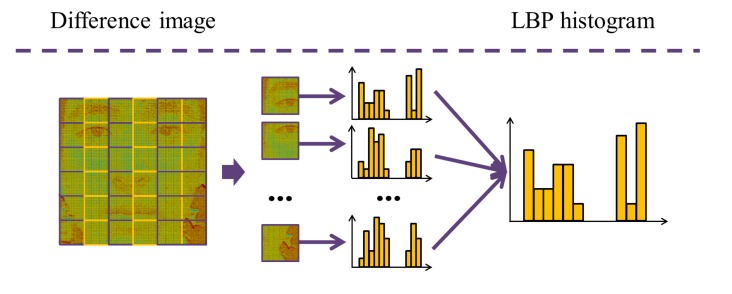
Process of extracting LBP histograms.

**Algorithm 2** Feature Extraction: Ray Difference Feature
1:Make 5 sub-aperture images, *SI_i_* (Figure 16)2:Normalize sub-aperture images, *nSI_i_* (Figure 17)3:Subtract *nSI_i_* from the center sub-aperture image *nSI_c_*4:Extract LBP histograms from the difference images (Figure 18)5:Concatenate LBP histograms (Figure 17)6:Apply PCA to the concatenated LBP histograms


At first, five sub-aperture images are made from the light field photograph. Figure 16 shows five sub-aperture images. Figure 16a is a sub-aperture image composed of center pixels of microlens images, and Figure 16b represents four sub-aperture images composed of adjacent pixels of microlens images. Five sub-aperture images have different viewpoints and information of light rays because different pixels are chosen in microlens images. In order to check the changes in lighting, we subtract the center sub-aperture image from adjacent sub-aperture images. In Figure 17, four difference images are depicted. Images in the first and second columns are normalized sub-aperture images. Face normalization [53] is the geometric normalization based on eye coordinates. Images in the third column are difference images between the center sub-aperture image and adjacent sub-aperture images. Depending on the directions of the incident rays, each difference image has diverse distributions. In order to analyze the ray difference, we extract local binary patterns (LBP) [51] from the difference image. Figure 18 illustrates the procedure of extracting LBPs from the difference image. The difference image is divided into several subregions with allowing the overlap between subregions. Each subregion yields one histogram. This histogram represents the distribution of uniform and rotation-invariant patterns at the local subregion. The dimensionality of the histogram is 59. In the final stage, we concatenate histograms extracted from four difference images as described in Figure 17. The dimensionality of the concatenated LBP histogram is 4 (= the number of the difference images) × *NP* (= the number of subregions per a difference image) × 59 (= the dimensionality of a LBP histogram). To reduce the dimensionality, PCA is applied.

### 3.2. Classification

**Figure 19.**
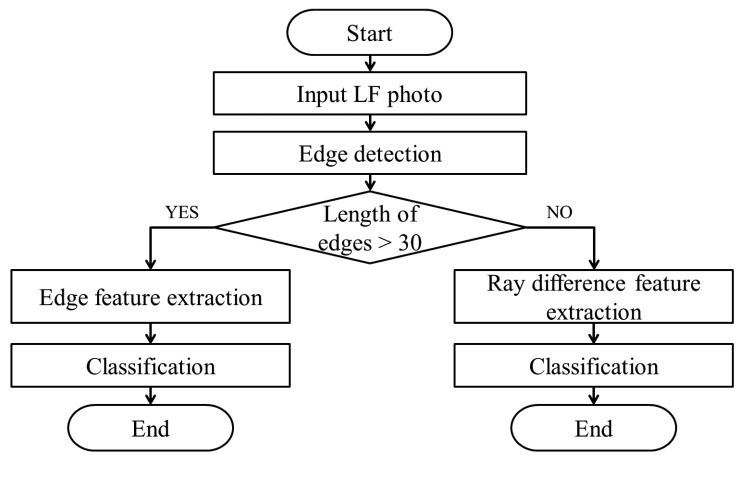
Process of classifying LF photographs with both edge and ray difference features.

We classify fake faces using edge and ray difference features. When both features are applied, the process of classification is a parallel structure as illustrated in Figure 19. According to the length of detected edges, which feature is extracted is determined. If the length of the edge is too short, it is difficult to decide whether the input face is real or not, because of the lack of the information. Therefore, if the quantity of the edge is not enough, we extract ray difference features and utilize them to distinguish fake faces. Support vector machine (SVM) is used for a classifier. Even though edge feature can be classified with a linear classifier, ray difference feature cannot. Radial basis function (RBF) is used as a kernel, and sigma values are adjusted depending on the distribution of features [54].

## 4. Experimentation and Discussion

### 4.1. Data Acquisition and Measures for Evaluation

There is no facial database which is taken by a light field camera. Thus, we collected light field photographs of real and fake faces with a Lytro camera [36]. The illuminating condition is indoor lighting. Types of spoofing attacks are listed in Table 1. There are three types of attacks with two different backgrounds. Attacks include the following:

- Normal print attacks (NP): 2D photos printed on A4 papers with a Fuji Zerox ApeosPort-II C5400 printer.
- Warped print attacks (WP): 2D photos, but they are bent over the face.
- HD tablet attacks (HD): high resolution screen attacks with an iPad 2. The resolution of an iPad 2 is 1024 by 768 pixels.

The background conditions include the following:

- Homogeneous background (HB): white and simple background.
- Complex background (CB): ordinary and indoor background.

**Figure 20.**
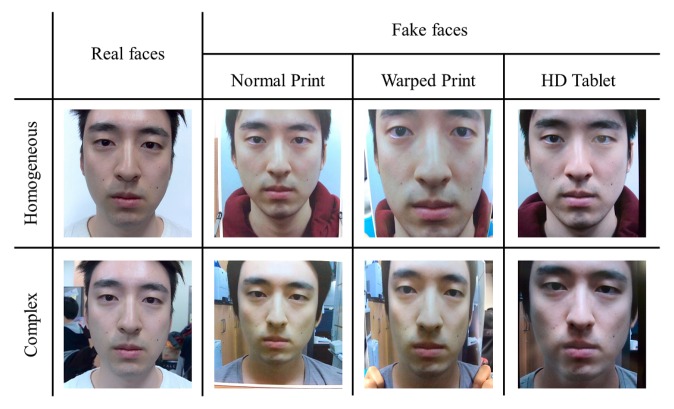
Samples of databases.

The number of light field photographs is listed in Table 2. The total number of subjects is 50. Light field photographs are decoded with Light Field Toolbox for Matlab [48]. Figure 20 shows samples of databases.

We evaluate the performance of our proposed method with our own databases. Databases are randomly categorized as 3 groups: training, development, and testing sets.

- Training set (30%): to be used for training the classifier.
- Development set (30%): to be used for estimating the threshold of the classifier.
- Testing set (40%): to be used for evaluating the performance.

Thirty percent of the subjects are used for training and development, and forty percent of the subjects are used for testing. Three groups are disjointed. That is, if images of subject A are used for training, they cannot be utilized for development or testing.

For numeric results, the following measures are used. Measures are expressed with terms in Table 3.

- False acceptance rate (*FAR*): the proportion of fake images misclassified as real. $\text{FAR}=\frac{FP}{FP+TN}$
- False rejection rate (*FRR*): the proportion of real images misclassified as fake. $\text{FRR}=\frac{FN}{TP+FN}$
- Total error rate (*TER*): the sum of FAR and FRR. *TER* = *FAR* + *FRR*
- Half total error rate (*HTER*): half of the TER. *HTER* = *TER*/2
- Accuracy: the ratio of the number of test images classified correctly and the total number of test images. $\text{Accuracy}=\frac{TP+TN}{TP+TN+FP+FN}$

### 4.2. Experiments and Results

**Figure 21.**
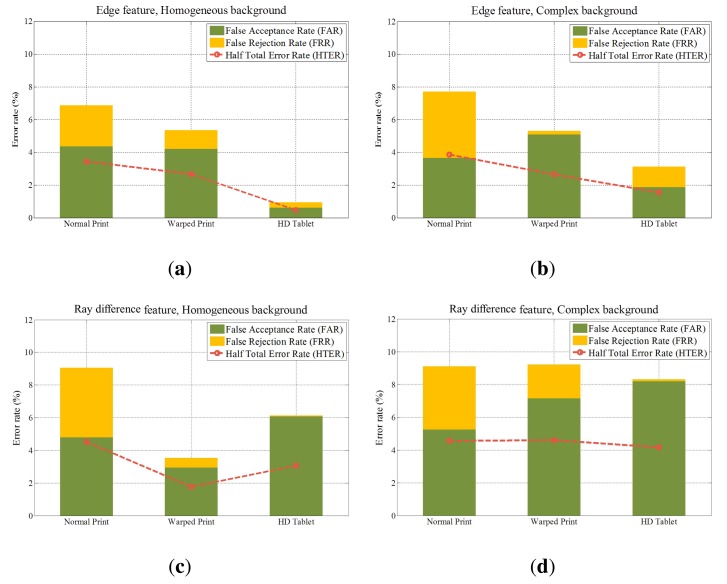
Error rates of (**a**) (Edge feature, Homogeneous background); (**b**) (Edge feature, Complex background); (**c**) (Ray difference feature, Homogeneous background); and (**d**) (Ray difference feature, Complex background).

We examine the performance in accordance with types of feature descriptors and spoofing attacks. Table 4 shows half total error rates (HTERs) of six types of fake faces. These numerical results are the averages of HTERs by carrying out experiments 10 times. Figure 21 presents false acceptance rates (FARs) and false rejection rates (FRRs) of each case.

**Figure 22.**
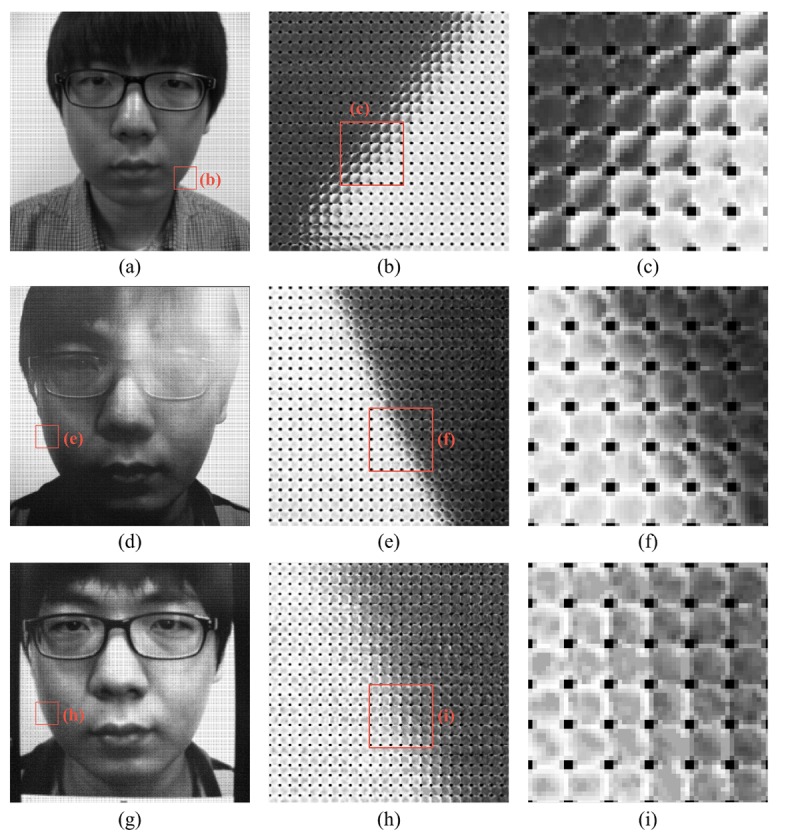
Comparison of light field photographs of a real face, a normal print and a HD tablet: (**a**) real face; (**b**) magnified view of (a); (**c**) magnified view of (b); (**d**) normal print; (**e**) magnified view of (d); (**f**) magnified view of (e); (**g**) HD tablet; (**h**) magnified view of (g); and (**i**) magnified view of (h).

Overall, edge feature has better performance than ray difference feature. Unlike ray difference feature, edge feature is extracted from the salient region (microlens images at defocused edge). Therefore, unnecessary information and noise in edge feature are relatively smaller than those in ray difference feature. Figure 22 illustrates light field photographs of a real face, normal print and HD tablet. In case of the real face, the light distribution inside the microlens (Figure 22c) is represented as the inverse of the macroscopic light distribution, as mentioned in Section 2.2.1. However, in the normal print and HD tablet light field photographs, the property of the microlens image is imperceptible. Moreover, the light distribution in the HD tablet light field photograph is more irregular than that in the normal print. HD tablet emits light autonomously, unlike normal prints and warped prints. Thus, not only reflected lights but also emitted lights are recorded by the light field camera. This widens the gap between edge features of the real face and fake face in the HD tablet. As a result, error rates under HD tablet attacks are smaller than other error rates. HTERs of edge feature under normal print attacks are 3.39% (homogeneous background) and 4.10% (complex background). Meanwhile, HTERs of edge feature under HD tablet attacks are 0.89% (homogeneous background) and 1.09% (complex background). Edge feature shows the strength against HD tablet attacks.

**Figure 23.**
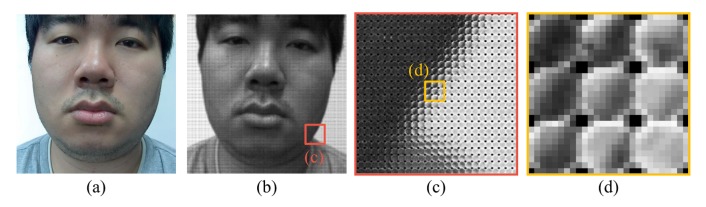
A light field photograph of a real face with a homogeneous background (**a**) a sub-aperture image; (**b**) a light field photograph; (**c**) a part of the light field photograph; and (**d**) enlarged microlens images.

**Figure 24.**
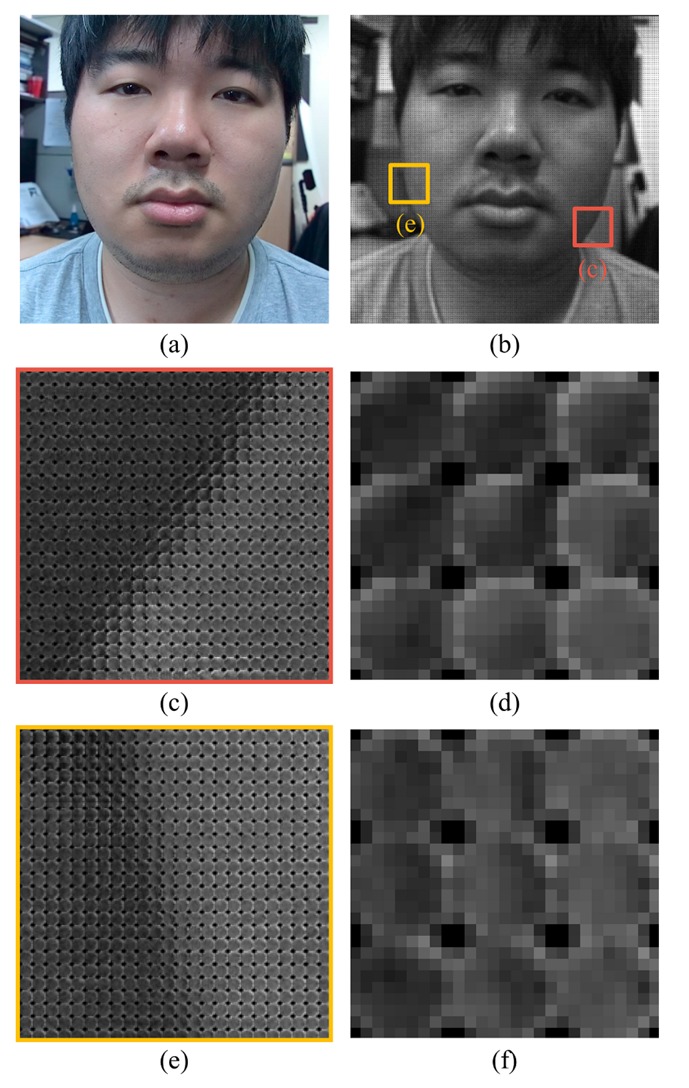
A light field photograph of a real face with a complex background (**a**) a sub-aperture image; (**b**) a light field photograph; (**c**) a part of the light field photograph (background: a skin-color locker); (**d**) enlarged microlens images of (c); (**e**) a part of the light field photograph (background: a gray partition); and (**f**) enlarged microlens images of (e).

**Figure 25.**
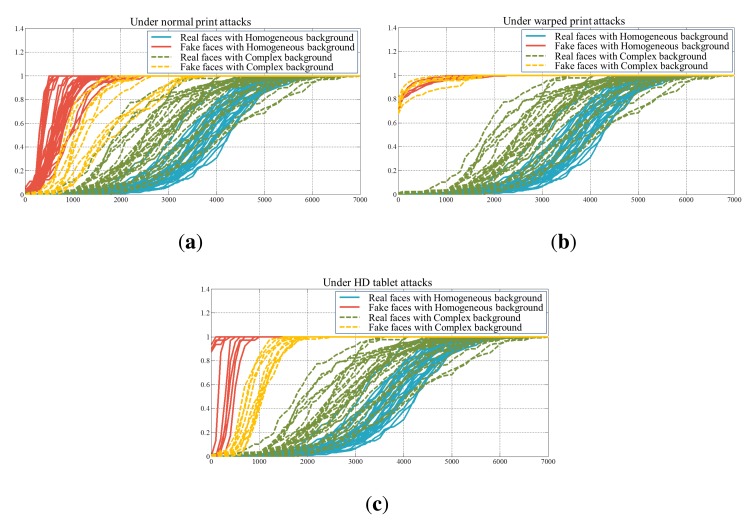
Cumulative distributions of variances (**a**) normal print; (**b**) warped print; and (**c**) HD tablet.

**Figure 26.**
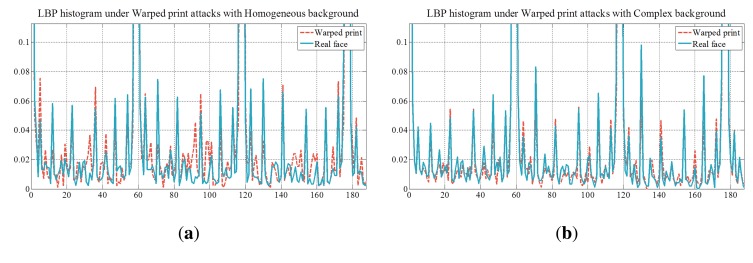
Local binary pattern histograms under warped print attacks (**a**) with Homogeneous background; and (**b**) with Complex background.

**Figure 27.**
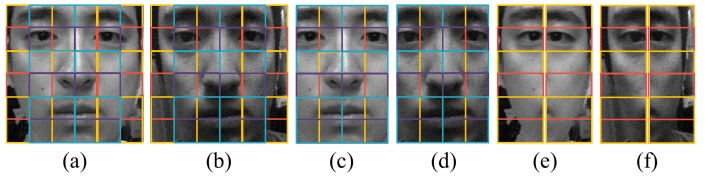
Subregions in the process of extracting a ray difference feature (**a**) a real face; and (**b**) a warped print attack including the complex background; (**c**) the real face; and (**d**) the warped print attack excluding the complex background; (**e**) removed subregions in the real face; and (**f**) removed subregions in the warped print attack.

The existence of the background also affects the performance. Figures 23 and 24 show microlens images of real faces with a homogeneous background and a complex background. When the background is homogeneous, the variation of the pixel values in a microlens image (Figure 23d) is large. However, the variation of the pixel values in a microlens image with the complex background (Figure 24d,f) is small. In Figure 24c,d, there is a locker whose color is similar to the skin color in the background. Also, when the intensities of the background and the skin are similar (Figure 24e,f), the pixel values in the microlens image are not discriminative. Due to the less variation of the pixel values in the microlens image with the complex background, it is more difficult to find the boundary between the face and the background in the microlens images, and variances of the microlens images are not so distinctive either. Figure 25 illustrates the cumulative distributions of variances under normal print, warped print, and HD tablet attacks. When the background is homogeneous, cumulative distributions of real and fake faces are discriminative (solid lines). Whereas, cumulative distributions of faces with the complex background are less distinguishable relatively (dotted lines). The low variances of microlens images with the complex background affect to the distributions of edge features, and this phenomenon results in the deterioration of the performance. In the case of the ray difference feature, complex background also deteriorates the performance under the warped print and HD tablet attacks, as illustrated in Figure 21. Figure 26 shows local binary pattern histograms of real faces and warped prints. These histograms are yielded during the procedure of extracting ray difference features in Figure 17. The background of faces in Figure 26a is homogeneous, and that of faces in Figure 26b is complex. Blue solid lines are LBP histograms of real faces, and red dotted lines are those of warped print attacks. When the background is homogeneous, the gap between LBP histograms of real face and warped print is large. However, the gap in the complex background is smaller than that in the homogeneous background. Because of the small gap, it is more difficult to discriminate real faces and warped prints. We can also check the influence of the background through an additional experiment. As described in Figure 27, we exclude the subregions that correspond to the background (Figure 27e,f). From subregions including the background (Figure 27a,b) and those excluding the background (Figure 27c,d), we extract ray difference features and classify the warped print attacks. When the subregions of the background are included in the process of extracting ray difference features (Figure 27a,b), the accuracy under the warped attack with the complex background (97.75%) is lower than the accuracy under the warped attack with the homogeneous background (99.22%). However, if the subregions of the background are excluded like Figure 27c,d, the performance is similar regardless of the type of the background (Table 5). Therefore, the performance under warped print attacks with the complex background is more deteriorated than that under warped print attacks with homogeneous background.

### 4.3. Discussion

In this paper, we propose a novel method for face liveness detection using characteristics of light field photograph. Then, our method cannot be applied to general face attack databases, such as Replay-Attack Database [55] and CASIA Face Anti-Spoofing Database [56]. Therefore, we choose the comparative method which can be applied to the light field database and which can utilize the representative characteristic of the light field photograph, refocusing technology.

**Figure 28.**
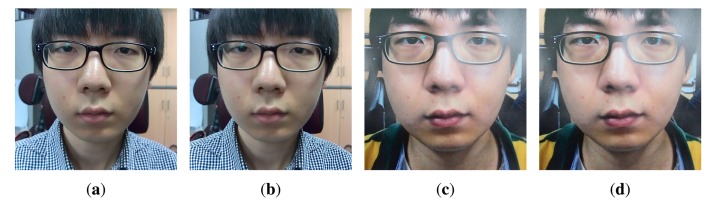
Refocused images (**a**) real face focused on the nose; (**b**) real face focused on the ear; (**c**) fake face focused on the nose; and (**d**) fake face focused on the ear.

Kim [27] proposed a countermeasure to spoofing attacks using variable focusing. Depending on the degree of defocussing, the depth between the nose and ears is estimated, and the defocus is used as a criterion to discriminate real and fake faces. Therefore, it is important to make the effect of defocussing great. This method requires two images whose focal planes are different. The focal plane of the first image lies on the nose tip, and that of the second image lies on the ears. In [27], those images are taken using a mirrorless camera. However, in this comparison, images are yielded from the light field photograph through the refocusing process. Refocusing means that users adjust the focal plane after taking pictures. This enables users to generate several refocused images from the only one light field photograph. Figure 28 illustrates refocused images made from the light field photographs. Figure 28a,b are real face images, and Figure 28c,d are fake face images. The focal plane of Figure 28a,c lies on the nose tip, and that of Figure 28b,d lies on the ears. With these refocused images, we conducted experiments for comparison with [27].

**Figure 29.**
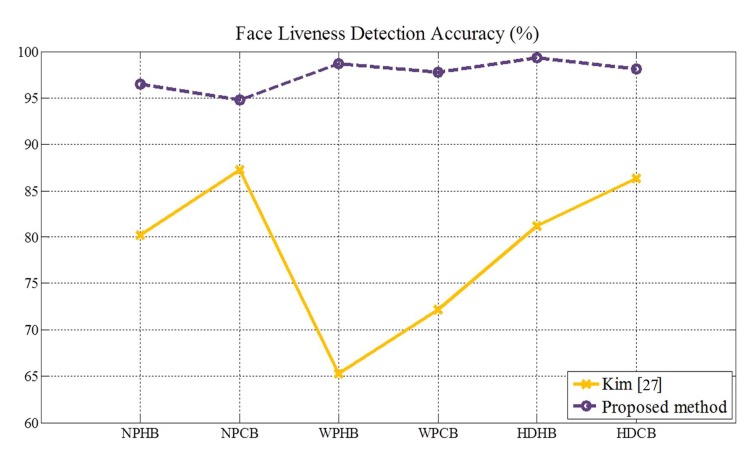
Face liveness detection accuracy (%).

Figure 29 and Table 6 present the results of two methods under normal print, warped print and HD tablet attacks. We computed the accuracies using both features. Like the process of classification explained in Section 3.2, we applied edge and ray difference features as the parallel structure, depending on the quantity of the edge. Our method acquires at least 94.78% accuracy or up to 99.36% accuracy. On the other hand, the best accuracy of [27] is 87.26%. The performance of our proposed method is superior to that of [27]. The reason why the performance of [27] is relatively low is that the effect of defocussing in refocused images is insignificant. In order that the remarkable degree of defocussing is obtained, depth of field (DoF), the range between the nearest and farthest objects in a given focal plane, must be sufficiently shallow, or the focal plane of the first refocused image must be far from the focal plane of the second refocused image. However, neither of the two conditions are satisfied. The light field camera can extend the DoF without decreasing the numerical aperture of the camera [50], but it is difficult to reduce the DoF. In addition, as the focal plane of the first image lies on the nose tip and that of the second image lies on the ears, the gap between the focal planes is not large, and DoFs are overlapped. Therefore, there is little difference between the two refocused images. This influences the performance of [27] to be deteriorated, and this is the reason why we avoid using refocused images for face liveness detection. Moreover, the performance of [27] under warped print attacks is the worst. This shows that [27], originally targeted at detecting 2D flat photo, is prone to warped print attacks. As a result, those accuracies are relatively low compared with other accuracies. Whereas, the performance of our proposed method is stable, regardless of types of spoofing attacks.

## 5. Conclusions and Future Work

We attempted to be the first to utilize raw light field photographs microscopically for detecting spoofing faces in this paper. Without taking image sequences, we can capture the change of the light distribution from only one light field data. Based on the characteristics of microlens image and sub-aperture image, new features, edge and ray difference features are developed. For evaluating our proposed method, we create a light field database with normal print, warped print and HD tablet attacks. The performance of the edge feature is superior to that of the ray difference feature. In particular, the usage of edge feature yields the best performance under HD tablet attacks (0.89% HTER in the homogeneous background and 1.09% HTER in the complex background). Moreover, comparing our method with another method [27], we show that our proposed method has good performance (96.51% accuracy under normal print attacks and 99.36% accuracy under HD tablet attacks in the homogeneous background).

Nowadays, the light field camera attracts engineers’ attentions. Although, in Section 4.3, we mentioned the limitation of using refocused images in order to discriminate the spoofing images with the comparative method [27], refocusing technology will be applicable to detect spoofing images by taking another approaches. Moreover, epipolar images [49], made from the raw light field photograph, can become a factor to estimate the depth of face. Likewise, there are many factors to apply the light field photograph to face liveness detection. In addition, light field camera modules, which can be embedded on cellular phones, have even been developed [38]. In future work, we will advance our method using that light field camera module and contribute to defending forged faces. In addition, by making up for the weakness at the complex background, we will make our system more robust to the practical environment. Furthermore, we will consider other countermeasures against evolved attacks such as videos and 3D masks by analyzing characteristics of the light field photograph.

## Figures and Tables

**Table 1.** Types of attacks (abbreviations).

	**Normal Print**	**Warped Print**	**HD Tablet**
Homogeneous Background	NPHB	WPHB	HDHB
Complex Background	NPCB	WPCB	HDCB

**Table 2.** Light field databases.

**Background**	**Real Faces**	**Fake Faces**		

		**Normal Print**	**Warped Print**	**HD Tablet**
Homogeneous	1104	766	250	500
Complex	1130	1066	250	500

**Table 3.** Measuring fake detection errors.

	**Object from Target (Real faces)**	**Object from Outlier (Fake faces)**
Classified as Target (Real faces)	True Positive (TP)	False Positive (FP)
Classified as Outlier (Fake faces)	False Negative (FN)	True Negative (TN)

**Table 4.** HTERs (%) in accordance with types of feature descriptors and spoofing attacks.

	**Edge Feature**			**Ray Difference Feature**		

	**Dev**	**Test**	**RBF Sigma**	**Dev**	**Test**	**RBF Sigma**
NPHB	3.48	**3.39**	0.68	4.94	4.13	5.2
NPCB	3.61	4.10	2.89	5.58	**3.53**	5.5
WPHB	2.87	**2.46**	0.43	0.62	2.93	25
WPCB	3.26	**2.03**	1.86	3.30	5.93	25.6
HDHB	0.05	**0.89**	0.3	3.63	2.50	25.8
HDCB	2.01	**1.09**	0.33	4.11	4.22	27.9

**Table 5.** Accuracy (%) of our proposed method with / without considering the background.

	**Including BG**		**Excluding BG**	

	**WPHB**	**WPCB WPHB**		**WPCB**
Accuracy	99.22	97.75	97.45	97.25

**Table 6.** Accuracy (%) and standard deviation of [27] and our proposed method.

	[27]	**Proposed Method**
NPHB	80.20 (3.58)	96.51 (1.54)
NPCB	87.26 (4.25)	94.78 (1.65)
WPHB	65.27 (2.23)	98.70 (0.90)
WPCB	72.21 (3.17)	97.73 (0.95)
HDHB	81.21 (2.35)	99.36 (0.63)
HDCB	86.35 (2.33)	98.10 (0.82)
